# A Review of the National Family Health Survey Data in Addressing India’s Maternal Health Situation

**DOI:** 10.3389/phrs.2022.1604825

**Published:** 2022-10-31

**Authors:** Papia Raj, Nilanjana Gupta

**Affiliations:** Department of Humanities and Social Sciences, Indian Institute of Technology Patna, Patna, India

**Keywords:** maternal health, systematic review, women, India, National Family Health Survey

## Abstract

**Objective:** This study aims to understand the trend of research conducted on issues of maternal health in India considering data provided in five rounds of National Family Health Survey (NFHS).

**Methods:** Systematic review of literature has been conducted using multi-stage search and review process adapted from Page et al.’s (2021) PRISMA. Initially 14,570 studies were identified and only 134 articles meeting selection criterion were considered in this study.

**Results:** Approximately 32% studies have focused on regional and state variation of maternal health status; while 27% dealt with utilization of maternal healthcare services; and 19% the socio-economic determinants of maternal health. While few studies have discussed the place of delivery, antenatal care and post-natal care visits, only five studies focus on issues related to women’s autonomy, including their health-seeking behaviour, knowledge, attitude and practices related to maternal health.

**Conclusion:** Non-communicable diseases and its role in maternal health still remains an unexplored domain of research on maternal health in India. Moreover, there exists geographical skewness in the number of studies conducted, focusing especially on few provinces while none on few others.

## Introduction

Maternal health, defined as the health of women during pregnancy, childbirth and the postpartum period, is an important indicator of development. Maternal mortality is considered to be a key measure of maternal health. As per the Sample Registration System (SRS) report by Registrar General of India (RGI), Maternal Mortality Ratio (MMR) in India was 113 per 100,000 live births during 2016–18, which is high compared to other developing nations. Some of the basic maternal health parameters include institutional deliveries, deliveries conducted by health personnel, antenatal care (ANC), postnatal care (PNC), and pregnancy outcomes [1]. There has been significant number of studies on maternal health and most of them have examined perspective of utilization and factors impacting maternal health. Few articles depicted background factors like education, maternal age, birth order, availability, accessibility and affordability of health care, wealth index, non-communicable diseases (NCDs), maternal infections, nutrition and lifestyle, strongly impact pregnancy outcomes in developing countries [2–7].

Millennium Development Goals (MDGs) adopted by United Nations in 2000 is one of the dominant reasons to focus research on maternal health. Out of the 18 goals, goal 5 has emphasised on improving maternal health. Later on, the failure of reaching MDG target instigated further research on maternal health. Apart from MDGs, few health policies and programmes in India, like National Health Mission (NHM), National Population Policy (NPP) in 2000 also retaliated discrepancies in maternal health and emphasised on reducing maternal mortality to improve maternal health. Sustainable Development Goals (SDGs) were launched in 2015 with broader goals and targets than MDGs. All these attracted large volume of research on maternal health in India. Thus, maternal health remains a major public health concern in India and there have been large disparities noticed at the provincial level as well as among various social groups.

Several health surveys are conducted in India, such as, Annual Health Survey (AHS), Indian Human Development Surveys (IHDS), District Level Household Survey (DLHS), National Family Health Survey (NFHS). These provide useful data on various indicators of maternal health. However, there is lack of consistency of data in these surveys. A study on review of national health surveys in India explicitly discussed that all these surveys cannot be compared due to several reasons [8]. First, not all indicators of maternal health were included in each survey. Second, many did not provide data for all provinces of India, for instance, AHS was conducted for the nine empowered action group (EAG) provinces. Third, none of these surveys have been conducted over same time period and many were discontinued after a couple of rounds of survey. Thus, a study suggested implementing one comprehensive survey that provides detailed information on relevant health indicators [9]. NHFS is considered to be robust and has been collecting data on all indicators of maternal health for entire geographical region of India since 1992 till 2021. Hence, in this review, studies based only on NFHS data have been analysed. There is a lack of review publications that collate evidences and highlight the issues on utilization of NFHS data for understanding maternal health situation.

Numerous studies have been published on maternal health. Most of the research have focused only on few aspects of maternal health such as, determinants and utilization of maternal health, delivery complications, etc. Though some health challenges like cultural issues, delaying obstetric care and service influences maternal health [10]. Either studies have been oriented only to examine ANC, PNC, and other determinants or conducted comparative regional analysis focusing on few provinces, lacking holistic approach to understand and analyse the scenario of maternal health in India. Therefore, through a systematic review of literature this study aims to focus on an overall understanding of which domains have been over-emphasised and which have been overlooked while discussing maternal health in India.

## Methods

**FIGURE 1:**
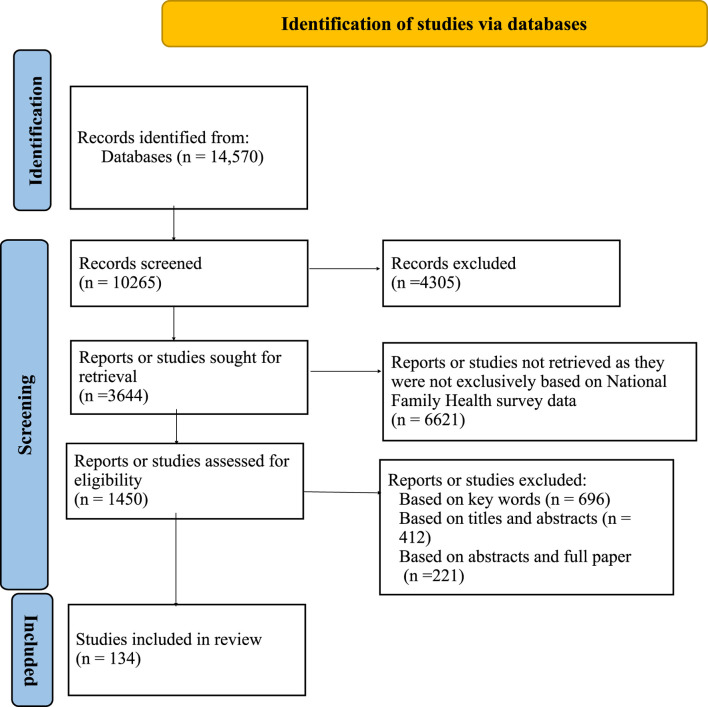
PRISMA flow diagram for articles selection (India, 1992–2022).

To proceed with systematic review approach, multi-stage search and review process from Page et al.’s (2021) PRISMA flow diagram as explained in Figure 1 was adopted. Literature was searched in organized steps on various online databases, including PUBMED, JSTOR, and Google Scholar. Initially 14,570 studies were identified. Due to the Covid pandemic, articles that are available only on digital platforms have been considered. Moreover, articles using specifically NFHS data have been examined in this study. NFHS is considered to be the Indian version of Demographic and Health Surveys (DHS). The first round of NFHS was conducted in 1992–1993, followed by four subsequent rounds- NFHS 2 (1998–1999), NFHS 3 (2005–2006), NFHS 4 (2015–16), and NFHS 5 (2019–2021).

After preliminary screening 10,265 studies were found and 4,305 studies were rejected based on several selection criterion. Studies that have used only NFHS data for analyzing maternal health and were published between 1992 and September 2022 are considered for this review. Based on this criterion 3,644 studies were sought for retrieval. Literature was searched and included in three stages. Total 1,450 studies were assessed for eligibility. In the first stage combinations of some generic keywords were used, such as, maternal health, NFHS, India. Few other words were added, like, utilization of maternal healthcare, maternal health outcomes (pregnancy), ANC, PNC, Iron Folic Acid (IFA) tablets consumption. In the second stage of literature search, to make the search more robust another set of keywords were included, for example, pregnancy, institutional delivery, maternal complications and morbidity, breastfeeding. Based on keywords, 696 studies were excluded. In the third stage title and abstracts of these articles were shortlisted. Then articles for which full texts were available were further reviewed. Thus, 412 studies were excluded based on titles and abstracts. While 221 studies were not included as full paper was not available. Using these inclusion and exclusion criterion, this paper is based on systematic review of 134 studies. Since only three studies have been published (until September 2022) using NFHS 5, therefore these three studies are not included in figures, as it would not depict the actual scenario.

## Results

**FIGURE 2:**
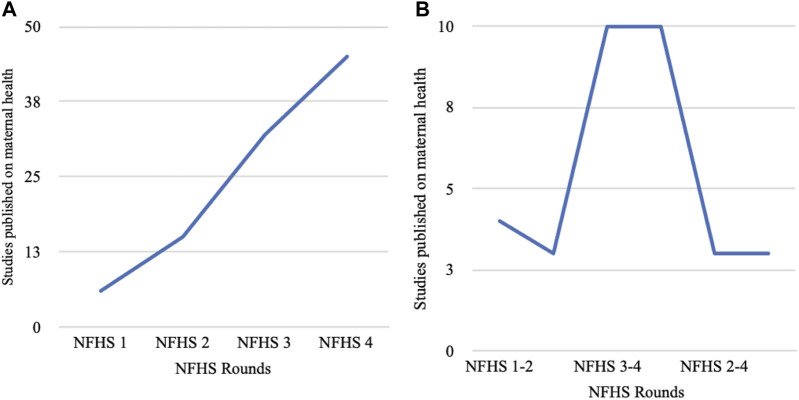
Studies published on maternal health using National Family Health Survey data (India, 1992–2022). **(A)** Studies based on single round of NFHS surveys. **(B)** Studies based on combined rounds of NFHS surveys.

All literature were arranged based on specific rounds of NFHS for which data had been considered in that study. This information enabled us to comprehend the trend of data analysis. Figure 2A (Studies published on maternal health using NFHS data) diagrammatically represents the trend of these data analysis. It is evident from the line graph that publications using NFHS data sets have increased since its inception. The peak in publication is noticed between NFHS 3 and NFHS 4. Considering the inclusive criteria, keywords and other factors, this review depicted only six studies on maternal health using NFHS 1, 15 studies using NFHS 2, 31, and 45 studies using NFHS 3 and NFHS 4 data, respectively. Only three studies have been reviewed using NFHS 5 data which is published until September 2022.

Certain contextual situations that shaped this trend with an increasing number of studies on maternal health can be explained by various changes in the policies on health, at global and national levels, that brought maternal health at forefront of health research. In the early 1990’s focus on reproductive and child health was not very profound in India. However, in the International Conference on Population Development (ICPD) in 1994 which also inspired NPP 2000 in India, women’s health was prioritized with special focus on reproductive and child health. This inspired academicians to conduct studies on these issues and maternal health being the most important component of reproductive health in India, surfaced as an integral part of such studies. Moreover, inception of the MDGs (2000) and SDGs (2015) listed improving maternal health as an important goal to achieve development. Thereafter several programs and policies that Indian government implemented implicated to improve maternal health and reduce maternal mortality. Due to this there has been a sharp increase in published articles from NFHS 3. This necessitated and encouraged more research on maternal health, especially analyzing trend and improvement in maternal health in India. Apart from policy changes, academicians and public health researchers in India got more familiarized with the rich source of NFHS data. In the initial rounds of NFHS researchers were still getting used to the enormous amount of information provided on maternal health on various indicators that were not earlier accessible in public domain. Nevertheless, over a period of time NFHS data were widely publicized and there was an increase in the amount of research conducted using these data sets.

### Indicators on RMNCH + A

This review has explored the perspective of maternal healthcare utilization based on five indicators (mothers who had full ANC, mothers who had three or more ANC, institutional delivery, skilled birth attendance, and PNC) given in RMNCH + A [1]. It is explicitly explained in Table 1, that 15 studies have specifically looked into all these five indicators of maternal health as per RMNCH + A, while ANC and institutional delivery have been specifically explored in nine and five studies, respectively. Among these, three studies were conducted using NFHS 3 [11–13], and five studies using NFHS 4 [14–18], other five studies using several combined rounds of NFHS [19–23]. It is evident from Table 1 that studies which have included PNC are mostly conducted during NFHS 3 and NFHS 4. However, it also needs to be noted that 17 studies have looked into the indicators of maternal health except for PNC. Out of 17 studies, four studies used NFHS 1 [24–27], and five studies used NFHS 2 [28–32], one study used NFHS 3 [33] and two used NFHS 4 data [34, 35]. Combined rounds of NFHS were used in four studies [36–39]. Five studies have specifically focused on institutional delivery including C-section delivery, in which three studies were conducted using NFHS 1 [40], NFHS 2 [41], and NFHS 4 [42], respectively. While two studies used both NFHS 4 and 5 [43, 44], nine studies have exclusively looked into ANC. Out of these nine studies, three studies are based on NFHS 2 [45–47], while one study on NFHS 3 [48] and four studies on NFHS 4 [49–52]. One study used NFHS 3–4 [53] for understanding the socioeconomic and geographical inequalities of ANC. The role of skilled birth attendees in maternal health has been examined in one study using NFHS 3 [54]. Two studies have focused on maternal health through the reproductive health index using NFHS 1 [24] and NFHS 3 [55] data. Two other studies have focused on pregnancy complications and adverse pregnancy outcomes on NFHS 4 [56] and all four rounds of NFHS [57].

**TABLE 1:** Frequency of studies based on specific maternal health indicators based on Reproductive, Maternal, Newborn Child plus Adolescent Health (India, 1992–2022).

Indicators	NFHS rounds									
	NFHS 1	NFHS 2	NFHS 3	NFHS 4	NFHS 3–4	NFHS 1–2	NFHS 2–4	NFHS 1–3	NFHS 1–4	NFHS 4-5
All five indicators of maternal health (RMNCH + A)	0	0	3	7	1	0	2	1	1	0
All indicators of maternal health except post-natal care	4	5	1	2	1	1	0	2	1	0
Institutional delivery (including C-section)	1	1		1	0	0	0	0	0	2
ANC (three or more and full)	0	3	1	4	1	0	0	0	0	0
Skilled birth attendees			1							
Maternal health through reproductive health index	1		1							
Pregnancy complications and adverse pregnancy outcomes				1					1	

### Age of Women

The review suggests that majority of articles include women belonging to 15–49 years. However, four studies [13, 14, 23, 33] have specifically analysed the age group of 15–19 years, other four studies examined 15–24 years [36, 55, 58, 59] and one study considered 20–24 years [60]. The age group of 15–24 years is considered to be vulnerable and maternal health behaviour at an early age ultimately affects pregnancy outcomes and reproductive health of women in later years. Studies on early pregnancy between 15–24 years have examined indicators like ANC and skilled birth attendees.

A study based on NFHS 1- NFHS 3 described the socio-economic disparities of maternity care among adolescent (15–19 years) women in India. It stated that proportion of adolescent women availing skilled birth attendees and full ANC has increased from 1990 to 2006 though 43% of women were married before the legal age [23]. Studies using NFHS 3 data on maternal care utilization among married adolescent (15–19 years) women depicted early childbearing and poor maternal health experiences are intimately linked to their educational and economic status [12, 33]. A similar study on NFHS 3 detected young women are less likely to have institutional delivery. Adolescent women (15–19 years), mostly in rural areas, lack autonomy and pregnancy related knowledge, resulting in lower maternal health service utilization [13].

Another study analysing four rounds of NFHS data (NFHS 1- NFHS 4) focused on maternal healthcare utilization in India among young women (15–24 years) [36]. This study contended that skilled birth attendees have increased since NFHS 1 among young women. However, a significant proportion of young women have not opted for full ANC during pregnancy. A study on mis-timed and unwanted pregnancies based on NFHS 3 found women in younger age group (15–24 years) are more likely to have mis-timed pregnancies whereas the likelihood of unwanted pregnancy is more among older women [59]. Another study on early marriage and its impact on reproductive health on NFHS 3 revealed women marrying early (before 18 years of age) were more likely to have poor reproductive health, experience child loss, and poor anthropometric indicators among children born [55]. This study has divided the age group into 10–19 years (adolescent), 20–24 years (young adulthood) and 25 years or older (adulthood). Lower education, poor living conditions, and less access to healthcare services result in adverse adolescent pregnancy outcomes [58]. Another study elaborated a significant association between unwanted pregnancies, pregnancy termination, and sterilization with lower age of marriage [60].

Against this backdrop, Figures 2A,B (Studies published on maternal health using various rounds of NFHS data) depicts the trend of how data from several rounds of NFHS were considered for understanding maternal health issues. This line graph is curvilinear in nature and shows nine studies have used NFHS 1–3 and NFHS 3–4. The publication increases in NFHS 3–4 and NFHS 1–3. However, only two studies have all four rounds of NFHS data. Majority of the articles have been published using NFHS 2–3 and NFHS 1–3. However, it should be noted that due to inclusion of new maternal health indictors in NFHS 4, sometimes research using combined rounds of all NFHS might be difficult. The following sections provide a nuanced analysis of maternal health conditions in each of the rounds of NFHS.

### National Family Health Survey 1 (1992–93)

Though only six studies were conducted on NFHS 1, yet they covered a range of issues. A study analysed the impact of caste on reproductive health variation among women and computed the Reproductive Health Index (RHI) in the eastern provinces of Bihar (including Bihar and Jharkhand), Orissa and West Bengal [24]. This study depicted caste to be an important predictor of reproductive health. Women belonging to upper caste are likely to have high RHI as compared to lower caste, due to better socio-economic status influencing their reproductive choices. Another study established similar findings, where women belonging to the scheduled caste (SC) are likely to have more complications due to caste influence on healthcare provisions [25]. Women belonging to economically, socially, and educationally weaker sections receive less maternal healthcare services in the southern states [26]. Education, parity, religion, caste, and place of residence impact breastfeeding activity of women [61]. Three articles have examined aspects of unmet need, breastfeeding, and C-section [14, 40, 62]. Maternal healthcare utilization is also influenced by type of health services [27]. However, there was major focus on various social determinants of maternal health, but family planning aspects, maternal mortality and morbidity were not covered in any of these studies. This might be attributed to the ongoing trend in research that gave more emphasis on factors influencing maternal health rather than outcome health status. There had been more emphasis on regional studies. Such studies allowed us to contextualize the broader framework for analysing maternal health scenario in India.

### National Family Health Survey 2 (1998–99)

Studies using NFHS 2 data on reproductive and maternal health have increased and included new aspects in research. Based on our inclusion criterion, 15 articles have primarily used NFHS 2 data for their analysis. It has been noticed that studies in NFHS 2 focused on ANC, PNC, service care utilization, C-section, and socio-economic determinants impacting maternal health. Out of 15 studies, seven studies have analyzed maternal healthcare utilization and socio-economic factors impacting maternal health [28, 29, 31, 32, 45, 47]. A study revealed that if socioeconomic and demographic factors were controlled then household type influences utilization of maternal health services [63]. This study stated women living in nuclear family have higher chances of utilizing maternal health services like ANC, PNC, etc. than those living in non-nuclear households. Another study was conducted in Bihar, Madhya Pradesh, Rajasthan, and Uttar Pradesh on ANC and indicated uneducated and poor pregnant women with at least one child were least likely to receive ANC and other services in these provinces [46]. Similar study was conducted in Uttar Pradesh looking into the pregnancy complications and health-seeking behaviour among married women. This study found nutritional status of women influences health problems during pregnancy [30]. Socio-demographic factors including place of residence, religion and caste, educational status of woman, woman’s work status, standard of living of the household, media exposure, age of mother at time of childbirth, birth order and birth interval impact pregnancy complications and their health seeking behaviour. Women belonging to rural areas and higher birth order have higher chances of delivering at home than in a public facility [41]. Factors, like availability of doctors, waiting time, cleanliness and affordability enhances the probability of reproductive healthcare uses [31]. It is evident from the above discussion that articles using NFHS 2 data have not examined aspects of family planning and role of skilled birth attendants. Compared to NFHS 1, there has been an increase in regional studies on maternal health, though there were no studies on provinces of Chhattisgarh, Goa, Gujarat, Haryana, Himachal Pradesh, Odisha, Sikkim, West Bengal, Punjab, and Tripura.

### National Family Health Survey 3 (2005–06)

There was a steady rise in number of publications based on NFHS 3 with a total of 31 articles. Three articles examined maternal healthcare utilization at provincial level [26–28] while two articles analysed child health and fertility [60, 64]. Like in the previous rounds of NFHS, in round three also some common areas have been focused on, such as ANC, PNC, safe delivery, C-section, role of economic factor and women’s autonomy. However maximum studies (23 studies) have pointed that there is a strong relationship between education and maternal health. Place of residence has emerged as another important factor in maternal health. In 2011 one study reported that women living in urban areas are more likely to use skilled birth attendants and stated about several financial, social, regional, and cultural barriers affecting availability and utilization of skilled birth attendant in India [54]. A study initiated that spousal violence likely affects maternal and child health (MCH) care. They depicted women experiencing any kind of spousal violence were less likely to use full ANC than those who did not experience any violence [35]. Women in the early ages are less likely to utilize maternal and child health services compared to those in the late age groups [33]. In a similar study it was established there has been a detrimental effect on reproductive health due to early marriage that often results in unplanned pregnancies, early motherhood, and abortions [55]. Articles using NFHS 3 data sets primarily focused on well-defined role of socio-demographic factors, regional differentials, nature of the household, ANC, C-section, spousal violence and women’s autonomy. Knowledge, attitude, adolescent motherhood, unwanted pregnancies and spousal violence were studied using NFHS 3 datasets which were not considered earlier.

### National Family Health Survey 4 (2015–16)

Based on inclusion criteria 45 articles on maternal health using NFHS 4 data were considered. It is evident from the published articles many common areas (such as determinants of maternal health, rural- urban differentials, and maternal health utilization, delivery care) have been over-emphasised like in the earlier rounds of NFHS. Articles have examined role of ANC and its influencing factors, C-section and regional disparities. Four studies have used spatial analysis in understanding maternal health care utilization [14, 15, 42, 65]. Studies established a strong association between mass media and maternal health in India [14–16, 42, 49, 56]. Usage of phone is positively associated with skilled birth attendance, PNC, usage of modern contraceptives but it is negatively associated with early ANC [17]. Utilization of Integrated Child Development Services (ICDS), registration of pregnancy and health insurance coverage increases the odds of full ANC utilisation [50]. Two studies highlighted men’s presence during ANC visits influences institutional deliveries [51, 66]. Poor sanitation practices adversely affect pregnancy outcomes [52]. Informed choice plays a pivotal role in use of modern contraceptive methods and may result in lower post-natal health problems, unmet need for contraceptives, unintended pregnancies, induced abortions, which adversely affect women’s health [34]. Articles have analysed role of women’s autonomy and found significant association with increasing odds of maternal healthcare services [18]. Decreased ANC, PNC, and institutional deliveries during Covid 19 pandemic are also noticed [67]. There have been considerable research in India on maternal health using NFHS 4 wherein socioeconomic factors along with utilization of maternal health care services have been overstated. New research areas like women’s autonomy, abortion, miscarriage and adverse pregnancy outcomes, use of contraception, role of skilled birth attendant was discussed in the studies based on NFHS 4. However, role of men and NCDs in maternal health need more attention for future research.

### National Family Health Survey 5 (2019–21)

The NFHS 5 data was released in May 2022, therefore only few studies have been published based on these data and only three studies have met the inclusion criteria. One of the studies analyzed changing scenario of C-section delivery [43]. It depicted that more than one-fifth of the institutional deliveries are C-section in most Indian provinces with maximum prevalence in southern provinces of Telangana, Kerala, and Andhra Pradesh. Another study was conducted on geographic and economic inequalities in C-section in the various districts of Bihar and reported that C-section delivery rate has increased from NFHS 4 to NFHS 5 [44]. Both these studies contend that increasing age of women, literacy of women, and economic strata are positively associated with C-section delivery in India [43, 44]. Critical appraisal of NFHS 5 data on maternal health indicator revealed prevalence of anaemia during pregnancy has increased from 15 to 17 provinces as compared to NFHS- 4 [68]. It was interesting to note that out of the three studies, two of them have emphasized on increasing C-section deliveries in India. Does this indicate that institutional delivery is promoting C-section? However, it needs to be mentioned that these three studies have used factsheets rather than raw data for analysis.

### Combined Rounds of National Family Health Survey

There are nine articles that have been published using NFHS round 1–3 and 11 using NFHS 3–4. However, only two articles have been published on maternal health using all four rounds of NFHS data [19, 36]. Study based on all the four rounds of NFHS datasets evaluated trends and determinants of maternal health care service utilization among young married women from 1992 to 2016 [36]. They stated that use of full ANC has increased from NFHS 1 to NFHS 4. Spousal violence has been an impounding factor impacting maternal health and studies show women experiencing physical violence from their husbands are less likely to adopt contraception and often experience unwanted pregnancy [57]. Several articles have inspected the differentials and determinants of maternal healthcare utilization [21, 22, 37–39, 53, 69]. A study scrutinized into this aspect using pooled data from 1998 to 1999 to 2015–2016, wherein illiteracy, women having five or more children, belonging to scheduled tribes, living in rural areas were associated with significantly low utilization of maternal health care services [21]. Two articles highlighted the importance of National Health Mission and Janani Suraksha Yojana for understanding maternal health in India [20, 70]. Articles published using data from NFHS 1–2 focused on broad aspects of breastfeeding, ANC- PNC care, and regional variation. A study based on NFHS 3–4 revealed socio-economic characteristics especially wealth status is a significant predictor of full ANC visits and institutional delivery in West Bengal [71]. Our review revealed that articles using more than one round of NFHS have basically analysed the change and trend of maternal health over the years focusing on time-series analysis.

**FIGURE 3:**
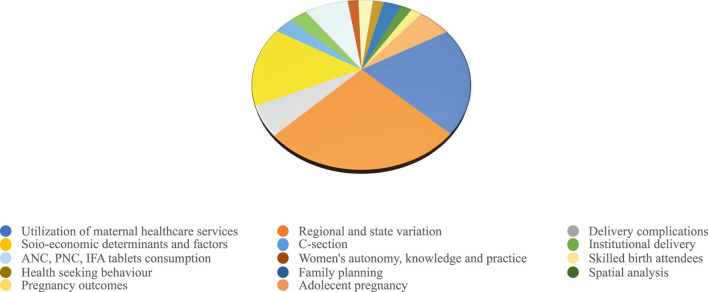
Areas focused on maternal health using National Family Health Survey data (India, 1992–2022).

Based on this systematic review a graphical representation about distribution of areas focused on maternal health has been presented in Figure 3 (Areas focused on maternal health using NFHS data). For preparing this pie diagram, the title and keywords of literature included in the review were considered. On basis of it, broad 15 categories of research areas were identified. Articles with common areas of interest have been included in multiple categories depending on their main focus. From the pie diagram it can be deduced that maximum articles have focused on regional and provincial variations (40 articles) of maternal health status followed by utilization of maternal healthcare services (32 articles). 24 studies have examined socio-economic determinants of maternal health. Eight studies have emphasised adolescent pregnancy, majorly on ANC and skilled birth attendees. This highlights the need to look into role of teenage and adolescent pregnancy and utilization of all-round maternal health services as well as pregnancy outcomes. It is evident from literature that socio-demographic factors are being illustrated in most of the studies either explicitly or indirectly. Delivery and pregnancy complications, ANC, PNC visits have been described in few studies. Women’s autonomy, particularly their role in maternal health, including their health-seeking behaviour, knowledge, attitude, and practices have been discussed in four to five articles. With new rounds of NFHS, spatial analysis is being used thoroughly. But articles considering knowledge, attitude and practices, pregnancy outcomes, role of men in maternal health, NCDs were scarce and needs more attention in future research. Therefore, this representative diagram gives us a snapshot of areas that have been researched since NFHS 1 till NFHS 4.

Table 2 (Geographical variation of areas focused on maternal health using NFHS data) shows the geographical variation of areas focused on maternal health using NFHS data. Studies covering entire country has increased since NFHS 1, the probable reason could be due to implication of policies and programmes focusing on maternal health. With the availability of district level data EAG provinces especially Bihar (14), Madhya Pradesh (9) and Uttar Pradesh (13) have been focused most. However, we could not find any study on Himachal Pradesh, Gujarat, Punjab. Among the North-Eastern provinces, Manipur (3) has maximum studies.

**TABLE 2:** Geographical variation of areas focused on maternal health using National Family Health Survey data (India, 1992–2022).

States	NFHS 1	NFHS 2	NFHS 3	NFHS 4	NFHS 5	NFHS 2 &3	NFHS 1–3	NFHS 1& 2	NFHS 3& 4	NFHS 2–4	NFHS 1–4	Total (n)
India	**3**	**6**	**17**	**31**	**2**	**2**	**7**	**3**	**6**	**3**	**2**	82
Andhra Pradesh	1	1	1	0	0	0	0	1	0	0	0	4
Arunachal Pradesh	0	1	0	1	0	0	0	0	0	0	0	2
Assam	0	0	0	1	0	0	0	0	0	0	0	1
Bihar	1	3	1	5	1	1	1	0	1	0	0	14
Chhattisgarh	0	0	0	2	0	0	1	0	0	0	0	3
Goa	0	0	0	0	0	0	0	0	1	0	0	1
Gujarat	0	0	0	0	0	0	0	0	0	0	0	0
Haryana	0	0	1	1	0	0	0	0	0	0	0	2
Himachal Pradesh	0	0	0	0	0	0	0	0	0	0	0	0
Jammu and Kashmir	0	0	0	1	0	0	0	0	0	0	0	1
Jharkhand	0	2	0	2	0	1	1	0	0	0	0	6
Karnataka	1	1	2	0	0	0	0	0	0	0	0	4
Kerala	1	2	0	0	0	0	0	0	1	0	0	4
Madhya Pradesh	0	2	1	5	0	0	1	0	0	0	0	9
Maharashtra	0	2	1	0	0	0	0	0	0	0	0	3
Manipur	0	1	0	2	0	0	0	0	0	0	0	3
Meghalaya	0	1	0	1	0	0	0	0	0	0	0	2
Mizoram	0	1	0	1	0	0	0	0	0	0	0	2
Nagaland	0	1	0	1	0	0	0	0	0	0	0	2
Odisha	1	0	0	1	0	0	0	0	0	0	0	2
Punjab	0	0	0	0	0	0	0	0	0	0	0	0
Rajasthan	0	2	1	1	0	0	0	0	1	0	0	5
Sikkim	0	0	0	0	0	0	0	0	1	0	0	1
Tamil Nadu	2	2	2	0	0	0	0	0	1	0	0	7
Telangana	0	0	0	0	0	0	0	0	0	0	0	0
Tripura	0	0	0	1	0	0	0	0	0	0	0	1
Uttar Pradesh	0	3	4	3	0	0	2	0	1	0	0	13
Uttarakhand	0	2	1	2	0	0	1	0	0	0	0	6
West Bengal	1	0	1	1	0	0	0	0	0	1	0	3

Bold indicate studies that covered entire India, while other studies have focused on particular provinces in India.

## Discussion

In India, maternal health is one of the pinnacles of public health discussions and debates by academicians, researchers, and policy makers. This review attempts to indicate the use of NFHS data for understanding maternal health situation in India. The findings suggest quite a lot of studies have been published on maternal health using NFHS datasets. Studies have analysed maternal health on wide spectrum at national, provincial, and district levels. Several aspects of maternal health, such as service utilization and health seeking behaviour (ANC, PNC, consumption of IFA tablets), regional variation, and role of socio-economic determinants have been studied excessively since the inception of NFHS 1 till NFHS 5. Few studies have examined unintended pregnancies and its impact on pregnancy outcomes as well as family planning methods. One of the most significant findings based on this systematic review is that out of 134 articles, 58 have highlighted the importance of education and its association in influencing maternal health. This reiterates that education is one of the most important social determinants, especially influencing maternal health. Literacy or community level education of pregnant mothers is important for understanding the effects of pregnancy, taking care of themselves, the role of midwives and nurses for experiencing healthy pregnancy [72]. This review has found that scholarly work using NFHS data has extensively exaggerated socio-economic and demographic factors, health seeking behaviour along with other co-health issues. There has been a focus to look into the geographical variation, in this EAG provinces have been emphasised the most. The review also highlighted that the role of NCDs on impacting maternal health, pregnancy complications have not been addressed rigorously, though few studies have focused on this issue partially. Therefore, this review will be insightful in the forthcoming research, so that more priority can be given in the domains that are having scarce research.
